# Effects of long-term fertilization on soil organic carbon mineralization and microbial community structure

**DOI:** 10.1371/journal.pone.0211163

**Published:** 2019-01-25

**Authors:** Zhen Guo, Jichang Han, Juan Li, Yan Xu, Xiaoli Wang

**Affiliations:** 1 Shaanxi Provincial Land Engineering Construction Group Co., Ltd., Shaanxi, Xi’an, China; 2 Institute of Land Engineering and Technology, Shaanxi Provincial Land Engineering Construction Group Co., Ltd., Shaanxi, Xi’an, China; 3 Key Laboratory of Degraded and Unused Land Consolidation Engineering, the Ministry of Land and Resources of China, Shaanxi, Xi’an, China; 4 Shaanxi Provincial Land Consolidation Engineering Technology Research Center, Shaanxi, Xi’an, China; 5 College of Agriculture, Guizhou University, Guizhou, Guiyang, China; Natural Environment Research Council, UNITED KINGDOM

## Abstract

Soil microorganisms play a pivotal role in carbon mineralization and their diversity is crucial to the function of soil ecosystems. However, the effects of long-term fertilization on microbial-mediated carbon mineralization are poorly understood. To identify the relative roles of microbes in carbon mineralization of yellow paddies, we investigated the long-term fertilization effects on soil properties and microbial communities and their relationships with carbon mineralization. The treatments included: no fertilization (CK), chemical fertilizer (NPK), organic fertilizer (M), and constant organic-inorganic fertilizer (MNPK). NPK treatment significantly increased soil water content (WC), while M and MNPK treatments significantly increased the content of soil organic carbon (SOC), total nitrogen (TN), soil microbial biomass carbon (SMBC), soil microbial biomass nitrogen (SMBN), and WC. Strong increases in CO_2_ emissions, potential mineralized carbon, and turnover rate constant were observed in both organic-fertilizer treatments (M and MNPK), relative to the CK treatment. These changes in soil properties can be attributed to the variation in microbial communities. NPK treatment had no significant effect. Different fertilization treatments changed soil microbial community; SOC and SMBN were the most important contributors to the variance in microbial community composition. The variations in community composition did not significant influence carbon mineralization; however, carbon mineralization was significantly influenced by the abundance of several non-dominant bacteria. The results suggest that SOC, SMBN, and non-dominant bacteria (*Gemmatimonadetes* and *Latescibacteria*), have a close relationship to carbon mineralization, and should be preferentially considered in predicting carbon mineralization under long-term fertilization.

## Introduction

Soil is the largest carbon pool in terrestrial ecosystems, and about 1.5 × 10^15^ kg of carbon is present in the form of soil organic matter. The carbon in soil organic matter is about three times the carbon storage of terrestrial vegetation [1], and a large proportion of the total CO_2_ emissions in the world comes from decomposition of soil organic matter [2]. The International Panel on Climate change (IPCC) [3] reported that agriculture is an important terrestrial ecosystem with the potential to mitigate the greenhouse effect. The natural potential of global agricultural greenhouse gas emission reduction is as high as 2300–6400 Mt CO_2_ equivalent per year, and more than 90% comes from reducing soil CO_2_ release. In the last 100 years, the increase in greenhouse gas concentrations caused by irrational farming practices and the massive burning of fossil fuels has changed the dynamic balance of carbon in the atmosphere [3–4]. The global surface temperature is expected to rise by 1.8–4.0 °C by the end of this century. Therefore, the study of soil organic carbon mineralization and its influencing factors has become one of the hot issues in global climate change.

Fertilization is a necessary agricultural farming method. Different fertilization methods lead to differences in soil nutrients, pH, and microbial species, which in turn affect the transformation and decomposition of organic carbon by soil microorganisms and, ultimately, greenhouse gas emissions from farmland [5]. Microbes are the main players in soil biochemical processes, and their abundance, activity, and composition are often closely related to soil organic carbon mineralization processes [6]. The application of chemical fertilizer has increased crop yield. For instance, Dai et al. [7] have shown that the long-term omission of N and P fertilizer significantly reduced wheat and maize yields, but chemical fertilizer also affects the biochemical processes of soil microbes. According to Eo and Park [8], long-term use of chemical fertilizers causes changes in bacterial community composition. For instance, Eo and Park [8] demonstrated that the *Chloroflexi* and *Planctomycetes* Phyla were negatively affected by chemical fertilization. Leff et al. [9] also showed that N and P in fertilizers caused shifts in microbial communities. On the other hand, Lee et al. [10] showed that the application of chemical fertilizers had no significant effect on soil microbial biomass carbon and CO_2_ emissions, which is primarily driven by microorganism composition, consistent with the results of Wagai et al. [11]. However, Dai et al. [12] showed that long-term application of chemical fertilizers can effectively increase CO_2_ emissions and is not directly related to soil microbial communities.

Regarding treatment with organic fertilizers, Grunwald et al. [13] and Ribeiro et al. [14] showed that the application of organic fertilizer promotes the rapid release of nutrients and significantly increases the mineralization of carbon pools. Organic carbon mineralization stems mainly from the decomposition of activated carbon pool components. Soil microbial biomass carbon and potential mineralized carbon are characterized by easy oxidation, easy decomposition, and easy mineralization. Soil microbial biomass carbon accounts for a small proportion of the soil organic carbon, which is more sensitive to the changes in soil quality and productivity than the total organic carbon [15]. Garcia-Pausas and Paterson [16] found that the mineralization of organic carbon was closely related to the community composition and abundance of microorganisms. Ramirez et al. [17] found that different fertilization treatments significantly change the composition of soil microbial communities, and lead to changes in the major bacteria in the soil. These studies suggest that differences in mineralization are driven by microorganisms. Furthermore, Zhang et al. [18] showed that interactions between soil microbial community composition and SOC, lead to changes in the dominant species in the microbial community, which in turn affect the turnover of SOC. However, the effects of fertilization treatments on microorganism-mediated alterations in the mineralization process is still unclear. Therefore, understanding the mechanism of microbial regulation of soil carbon pool mineralization is of great significance for the accumulation and sustainable development of farmland soil carbon pool.

As the only province in China with no plain support, Guizhou Province is dominated by mountains and hills. With an increasing population, nonagricultural land use is increasing and agricultural land is gradually decreasing. Therefore, investigations into fertility conservation and sustainable use of existing cultivated soil are urgently needed. As an important soil resource, paddy soil from yellow earth accounts for 46% of the paddy soil area in Guizhou Province and has a large production function. In addition, yellow paddy soil also has an ecological function of fixing atmospheric CO_2_ and mitigating global climate change. Previous studies have focused on the biological activity, physicochemical properties, and carbon and nitrogen balance of yellow paddy soil [19,20]. However, mineralization as a process of carbon output has not been studied in yellow soil. Furthermore, the impact of chemical fertilizer application on organic carbon mineralization has not been clearly defined, and the contribution of soil microbes to soil carbon pool change under different long-term fertilization conditions are still unclear.

The objectives of this study were to (1) study the effects of different fertilization measures on soil properties, microbial community abundance, and composition; (2) clarify the effects of different fertilization treatments on soil organic carbon mineralization; and (3) clarify the relative relationship between carbon mineralization parameters, soil properties, and soil microbial communities in paddy soils. We hypothesized that (1) the organic fertilizer treatment will significantly increase organic carbon mineralization and turnover rate compared to chemical fertilizer treatment; and (2) the main dominant species of bacteria will have a lower relative effect on organic carbon mineralization than other species under different long-term fertilization treatments.

## Materials and methods

### Study site

The long-term experimental site was located in Guizhou Academy of Agricultural Sciences (106°39'E, 26°29'N). Seated in the hilly area of central Guizhou, the study site has a subtropical monsoon climate with an average elevation of 1071 m and average annual temperature 15.3 °C. The average annual sunshine duration is about 1354 h and the relative humidity is 75.5%. The annual frost-free period is about 270 days and the annual precipitation is 1100–1200 mm. The soil type of the experimental site is yellow paddy soil, and the parent material is Triassic limestone and sand shale weathering material. The soil is sticky, heavy, and has a high silt content. Iron-manganese bound or red-white interbedded layer, which lack organic matter and easily harden, appear in the shallow layer at some places. Due to an insufficient water source, the area is vulnerable to drought disaster. A yearly rice crop was grown in the experimental fields for most of the experimental period, with a period of 5 years (from 2002 to 2006) of corn crop.

### Experimental design

The long-term location test began in 1995 and the data was collected in 2016. Thus, the study investigates the effects of fertilization treatments over 22 years in a field. The experiment was carried out over a large area with consistently separate treatment groups without any overlap. The plot area of 201 m^2^ (35.7 m × 5.6 m) was divided into 4 treatment areas. Four representative fertilization treatments were selected for study on the long-term experimental site: no fertilization (CK), chemical fertilizer (NPK), organic fertilizer (M) and constant organic-inorganic fertilizer (MNPK). The chemical fertilizer (NPK) included urea (including N 46%), superphosphate (including 16% P_2_O_5_), and potassium chloride (including 60% K_2_O). For the NPK group, 2–3 days before annual rice transplanting in April, 50% of the urea was applied first followed by superphosphate and potassium chloride, as the base fertilizer. The remaining 30% and 20% of the urea were applied during two topdressing periods, 7–10 days after transplanting the rice, and then later in the growing season, respectively. The application amount of chemical nitrogen fertilizer (NPK) was adjusted each year according to the nutrient content of organic fertilizer. The organic fertilizer was cattle manure (including C 413.8 g kg^-1^, N 2.7 g kg^-1^, P_2_O_5_ 1.3 g kg^-1^, K_2_O 6.0 g kg^-1^). Annual application of organic fertilizer consisted of 61.1 t ha^-1^ for the M treatment. The NPK treatment consisted of N 165 kg ha^-1^, P_2_O_5_ 82.5 kg ha^-1^, and K_2_O 82.5 kg ha^-1^. M fertilizer was applied at one time, 2–3 days before rice transplanting, as a base fertilizer. The M treatment did not require topdressing. In the MNPK treatment, the superphosphate and potassium chloride and part of the urea, (50%) were applied as a base fertilizer before the transplanting. The remaining 30% and 20% of the urea were applied during two topdressing periods, 7–10 days after transplanting the rice, and then later in the growing season, respectively.

### Soil sample collection

Rice was harvested once a year. The rice was transplanted in April each year, and harvested in mid-to-late October; the soil rested for approximately 6 months after harvest, i.e. no crops were planted until the following year. Samples were collected within one week after the rice was harvested in mid-to-late October 2016. At this stage, the surface soil changes from anaerobic to aerobic respiration, and soil moisture is not a dominant factor. Therefore, the correlation between soil organic carbon mineralization and the microbial community was studied in this context to understand soil changes during the fallow period.

Each of the 4 treatment areas was equally divided into 3 sections (67 m^2^). Samples were collected from 5 evenly spaced spots from each section, and then combined to get a representative sample. Three soil samples were collected from each treatment area. After collection, soil samples were stored in a cooler (for up to 4 hrs) and transported back to the laboratory. After sieving (2-mm) to remove rice root residue and animal residues, the sample was divided into three parts. One part was frozen at -80 °C for high through put sequencing analysis, one part was stored at 4 °C until determination of soil microbial biomass and water content, and the remaining part was air dried naturally to measure soil pH, total nitrogen, and organic carbon.

### Soil chemical analysis

Soil samples were air-dried at room temperature for approximately 15 days. After drying, the samples were sieved (< 2 mm), and then suspended in water at a soil-to-water ratio of 1:5. Suspensions were mixed end-over-end for 1 h at 25 °C, then pH was measured using a pH meter (PHS-3E, INESA, China) [21]. The soil organic C and total N were determined by dichromate oxidation and Kjeldahl digestion, respectively [22]. Soil water content was measured gravimetrically after drying at 105°C for 24 h. Soil microbial biomass C and N were determined using the chloroform fumigation-extraction method [23–24].

### Organic carbon mineralization

Organic carbon mineralization was measured using the lye absorption method for 30 days [14]. Thirty grams of soil was adjusted to 60% of field capacity with deionized water and placed in a 50 mL beaker. The 50 mL beaker was placed in the bottom of a 1000 mL culture flask, and pre-cultured for 1 day at 25 ± 1 °C. A 50 mL absorption cup containing 10 mL of 0.1 mol L^-1^ NaOH solution was placed on the bottom of the flask. After sealing the flask, the sample was incubated in a dark incubator at 25 ± 1 °C. On the 3rd, 6th, 9th, 12th, 15th, 18th, 21st, 24th, 27th, and 30th day of culture, the lye absorption cup was replaced. Water was added to the soil to constant weight each time the lye absorption cup was changed. After addition of 2 mL of 1 mol L^-1^ BaCl_2_ solution and 2 drops of phenolphthalein indicator, the samples were titrated to a colorless solution with 0.1 mol L^-1^ HCl (calibrated with borax before each titration). The cumulative mineralization amount of organic carbon was calculated based on the amount of CO_2_ released during the culture period. The Ct value refers to the total amount of soil CO_2_ release from the start of the cultivate to a certain point time. For example, the cumulative mineralization amount on the 9th day is equal to the sum of the mineralization amounts of the 3rd day + the 6th day + the 9th day. Soil organic carbon mineralization (CO_2_ mg kg^-1^) = C_HCl_ × (V_0_ –V_1_) × 22 / 0.03 at each time point; where C_HCl_ is the concentration of hydrochloric acid, mol L^-1^; V_0_ is the volume of the blank titration, mL; V_1_ is the volume of hydrochloric acid consumed, mL.

### High-throughput sequencing

Microbial DNA was extracted from 0.5 g of soil samples using the E.Z.N.A. soil DNA Kit (Omega Bio-tek, Norcross, GA, USA) according to the manufacturer’s protocol. The final DNA concentration and purity were determined using a NanoDrop 2000 UV-vis spectrophotometer (Thermo Scientific, Wilmington, USA). DNA quality was checked by 1% agarose gel electrophoresis. The V3-V4 hypervariable regions of the bacteria 16S rRNA gene were amplified using a thermocycler PCR system (GeneAmp 9700, ABI, USA) and the following primers: 338 F (5’-ACTCCTACGGGAGGCAGCAG-3’) and 806R (5’-GGACTACHVGGGTWTCTAAT-3’) [25]. The PCR reactions were conducted using the following program: 3 min of denaturation at 95 °C, 27 cycles of 30 s at 94 °C, 30s for annealing at 55 °C, and 45s for elongation at 72 °C, and a final extension at 72 °C for 10 min. PCR reactions were performed in triplicate. The 20 μL PCR mixture contained the following: 4 μL of 5 × FastPfu Buffer, 2 μL of 2.5 mM dNTPs, 0.8 μL of each primer (5 μM), 0.4 μL of FastPfu Polymerase, and 10 ng of template DNA. Three samples were selected for PCR amplification in each treatment. The resulting PCR products of the same treatment were mixed, extracted from a 2% agarose gel, and further purified using the AxyPrep DNA Gel Extraction Kit (Axygen Biosciences, Union City, CA, USA).

Purified amplicons were pooled in equimolar portions and paired-end sequenced on an Illumina MiSeq platform (Illumina, San Diego, USA) according to the standard protocols by Majorbio Bio-Pharm Technology Co. Ltd. (Shanghai, China).

### Date analysis

Raw fastq files were quality-filtered by Trimmomatic [26] and merged by FLASH [26] with the following criteria: (1) Reads were truncated at any site receiving an average quality score < 20 over a 50 bp sliding window; (2) Sequences whose overlap were longer than 10 bp were merged according to their overlap with no more than 2 bp mismatch; (3) Sequences of each sample were separated according to barcodes (exactly matching) and primers (allowing 2 nucleotide mismatching), and reads containing ambiguous bases were removed.

UCLUST was used to cluster high-quality sequences into operational taxonomic unit (OTU) with thresholds set at 97% [27]. The RDP-classifier Bayesian algorithm [24] was used to annotate the 97% similar OTU representative sequences to obtain taxonomic information for each OTU. Sequences that could not be clustered in any taxonomically known group were defined as unclassified [28].

The first-order kinetic equation was used to fit the cumulative mineralization of soil organic carbon by Origin 9.0 [14],

$${\text{C}}_{\text{t}}={\text{C}}_{0}(1-{\text{e}}^{-\text{kt}})$$ (1)

Where: C_t_ is the cumulative C mineralization of soil after t time, g kg^-1^; C_0_ is the potential mineralizable carbon in soil, g kg^-1^; k is the turnover rate constant of organic carbon pool, d^-1^; t is the number of days of cultivation, d; Half cycle T_1/2_ = ln2/k.

The soil basic physicochemical properties and mineralization parameters were compared using ANOVAs with Microsoft Excel 2010 and SPSS 20.0 software. Multiple comparison posthoc analyses were performed using the Duncan method to test for significant differences.

The bacteria community composition and abundance at the Phylum level for each sample was calculated using R 3.3.2 software. Pearson correlations were used to compare basic soil properties (Table 1) and mineralization parameters (Table 2) in Table 3, and to compare environmental factors and bacterial abundance to generate the correlation matrix heatmap diagram. Principal Component Analysis (PCA) was performed to analyze the bacterial community composition and environmental factors on the Phylum level. This data was analyzed using the free online platform of Majorbio I-Sanger Cloud Platform (www.i-sanger.com).

**Table 1:** Basic properties of soil samples under different fertilizer treatments.

Treatment	SOC (g kg^-1^)	TN (g kg^-1^)	SMBC (mg kg^-1^)	SMBN (mg kg^-1^)	pH	WC (%)	qMB (%)	SMBN/TN (%)
CK	25.35 ± 1.53 b	2.27 ± 0.23 b	446.13 ± 35.14 c	28.28 ± 2.62 c	7.14 ± 0.04 ab	26.46 ± 1.79 b	1.78 ± 0.33 b	1.27 ± 0.40 b
NPK	26.66 ± 1.61 b	2.03 ± 0.25 b	478.96 ± 39.56 c	41.91 ± 3.70 bc	7.06 ± 0.14 b	31.58 ± 2.15 a	1.80 ± 0.43 b	2.09 ± 0.82 ab
M	41.44 ± 1.20 a	2.68 ± 0.25 a	837.84 ± 69.17 b	73.50 ± 6.75 a	7.00 ± 0.23 b	32.36 ± 2.90 a	2.02 ± 0.12 b	2.76 ± 0.52 a
MNPK	37.99 ± 1.41 a	3.02 ± 0.12 a	1243.75 ± 103.58 a	58.43 ± 5.60 ab	7.50 ± 0.29 a	32.33 ± 1.47 a	3.28 ± 0.17 a	1.93 ± 0.19 ab

Notes: Different small letters indicate significant differences (P < 0.05) within the same column. CK: no fertilization; NPK: chemical fertilizer; M: organic fertilizer; MNPK: constant organic-inorganic fertilizer. SOC, total soil organic carbon; TN, total nitrogen; SMBC, soil microbial biomass carbon; SMBN, soil microbial biomass nitrogen; WC, soil water content; qMB, ratio of soil microbial biomass carbon to soil organic carbon; SMBN/TN, ratio of soil microbial nitrogen to total nitrogen in soil. (n = 3, mean ± SE)

**Table 2:** Model parameters and coefficients of determination (*R*^2^) estimated using the first-order exponential model fitted to the cumulative C mineralized data from the different treatments.

Treatment	C0 (g kg^-1^)	k (d^-1^)	T_1/2_ (d)	C_0_/SOC (%)	*R^2^*
CK	1.23 b	0.032 c	21.7 a	4.8 a	0.998 **
NPK	1.26 b	0.048 ab	15.4 c	4.7 a	0.998 **
M	2.10 a	0.047 a	14.8 c	5.1 a	0.996 **
MNPK	2.62 a	0.039 bc	17.8 b	6.9 a	0.998 **

Notes: CK: no fertilization; NPK: chemical fertilizer; M: organic fertilizer; MNPK: constant organic-inorganic fertilizer. C_0_, amount of potential mineralizable SOC; k, constant of mineralization rate of SOC; T_1/2_, half turnover period; C_0_/SOC, ratio of potential mineralizable organic carbon to total organic carbon in soil. Different small letters indicate significant differences (P < 0.05) within the same column.

**, statistically significant (P < 0.01)

**Table 3:** Pearson correlation coefficients between mineralization parameters and basic soil properties.

Item	SOC	TN	SMBC	SMBN	pH	WC	qMB	SMBN/TN
C_t_	0.8786*	0.5614	0.4138	0.9434*	0.9289*	0.4822	0.9998 **	0.0406
C_0_	0.9735**	0.8869*	0.7756	0.9986**	0.6502	0.6537	0.8858 *	0.4386
k	0.9201*	0.7885	0.8602	0.9388*	0.3518	0.9058*	0.6837	0.7688
T_1/2_	-0.8623	-0.7072	-0.8257	-0.9006*	-0.3325	-0.9560*	-0.6619	-0.7725
C_0_/SOC	0.5609	0.8857*	0.9435*	0.4026	0.9376*	0.4474	0.9985 **	0.0231

Notes: C_0_, amount of potential mineralizable SOC; k, constant of mineralization rate of SOC; T_1/2_, half turnover period; C_0_/SOC, ratio of potential mineralizable organic carbon to total organic carbon in soil. SOC, total soil organic carbon; TN, total nitrogen; SMBC, soil microbial biomass carbon; SMBN, soil microbial biomass nitrogen; WC, soil water content; qMB, ratio of soil microbial biomass carbon to soil organic carbon; SMBN/TN, ratio of soil microbial nitrogen to total nitrogen in soil.

* indicates P < 0.051,

** indicates P < 0.01.

Analysis of Similarities (ANOSIM) was used to determine if differences between groups were greater than intragroup differences. The data were analyzed on the free online platform of Majorbio I-Sanger Cloud Platform (www.i-sanger.com).

## Results

### Basic soil properties

The results in Table 1 showed that long-term fertilization treatments significantly affected the basic soil properties. NPK treatment only induced significantly increased WC compared to CK; all other environmental factors did not change significantly. Treatment with organic fertilizer (M and MNPK), caused significantly increased SOC, TN, SMBC, SMBN, and WC by 63.47% and 49.85%, 18.01% and 33.2%, 87.80% and 178.79%, 159.90% and 106.62%, and 22.28% and 22.18%, respectively, in M and MNPK groups compared to no fertilizer (CK). The qMB also increased significantly in the MNPK group compared to all other groups. SMBN/TN increased significantly in the M group only compared to the CK group. The pH increased in the MNPK group compared to the NPK and M groups, but was not significantly different from the CK group.

### Soil organic carbon mineralization

**Fig 1:**
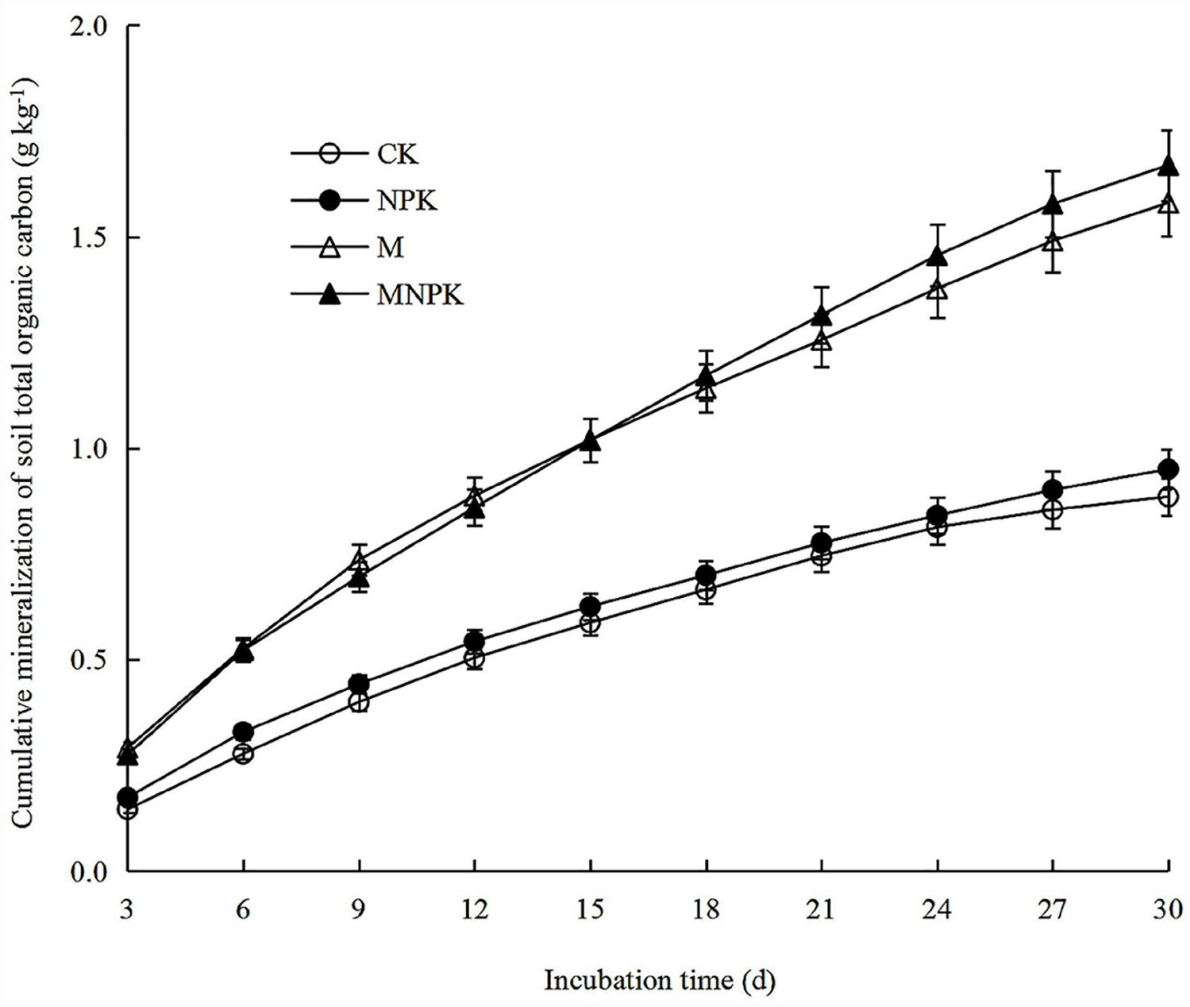
Cumulative carbon mineralization during 30 days of incubation. Data points represent averages ± standard errors (n = 3).

The CO_2_ cumulative release increased with culture time under various fertilization treatments, but the cumulative release intensity gradually decreased (Fig 1 and S1 Table). The cumulative organic carbon mineralization across the entire incubation period ranged from 0.15 to 1.67 g kg^-1^ among all fertilization treatments. Cumulative mineralization of soil total organic carbon was significantly higher with organic fertilizer treatment (M and MNPK), compared to NPK and CK treatments. No significant differences between MPK and CK treatments were observed. At the end of the culture, the cumulative organic carbon mineralization in NPK, M, and MNPK treatment increased by 7.4% (P > 0.05), 78.4% (P < 0.05), and 88.5% (P < 0.05), respectively, compared to CK.

### Modeling of the C-mineralization dynamics

Results from fitting the kinetic model to mineralization data are shown in Table 2. In the case of all treatments (CK, NPK, M and MNPK), the model values of R^2^ were very similar (P < 0.01): 0.998, 0.998, 0.996, and 0.998 in CK, NPK, M, and MNPK treatments, respectively. C_0_ indicates the amount of potential mineralizable organic carbon. The application of organic fertilizer (M and MNPK) was more effective than chemical fertilizer at altering C_0_; MNPK and M increased significantly by 113.01% and 70.73%, respectively, while NPK was not significantly different from no fertilizer treatment (CK). The incubation time required to mineralize half of the C_0_-half-life time (T_1/2_) was 15.4 d for NPK, 14.8 d for M, and 17.8 d for MNPK treatment. T_1/2_ in all three treatments were significantly less than no treatment (CK). The order of k values were NPK > M > MNPK > CK. There were no differences in C0/SOC, the ratio of potential mineralizable organic carbon to total organic carbon in soil.

Table 3 shows the Pearson correlation between mineralization and basic soil properties. C_t_ significantly positively correlated with SOC, SMNN, pH and qMB. C_0_ significantly positively correlated with SOC, TN, SMBN and qMB. The k significantly positively correlated with SOC, SMBN, and WC. T_1/2_ significantly negatively correlated with SMBN and WC, and C_0_/SOC was significantly positively correlated with TN, SMBC, pH, and qMB. These results indicate that the C mineralization parameters were closely related to the basic soil properties.

### Bacterial community composition

**Fig 2:**
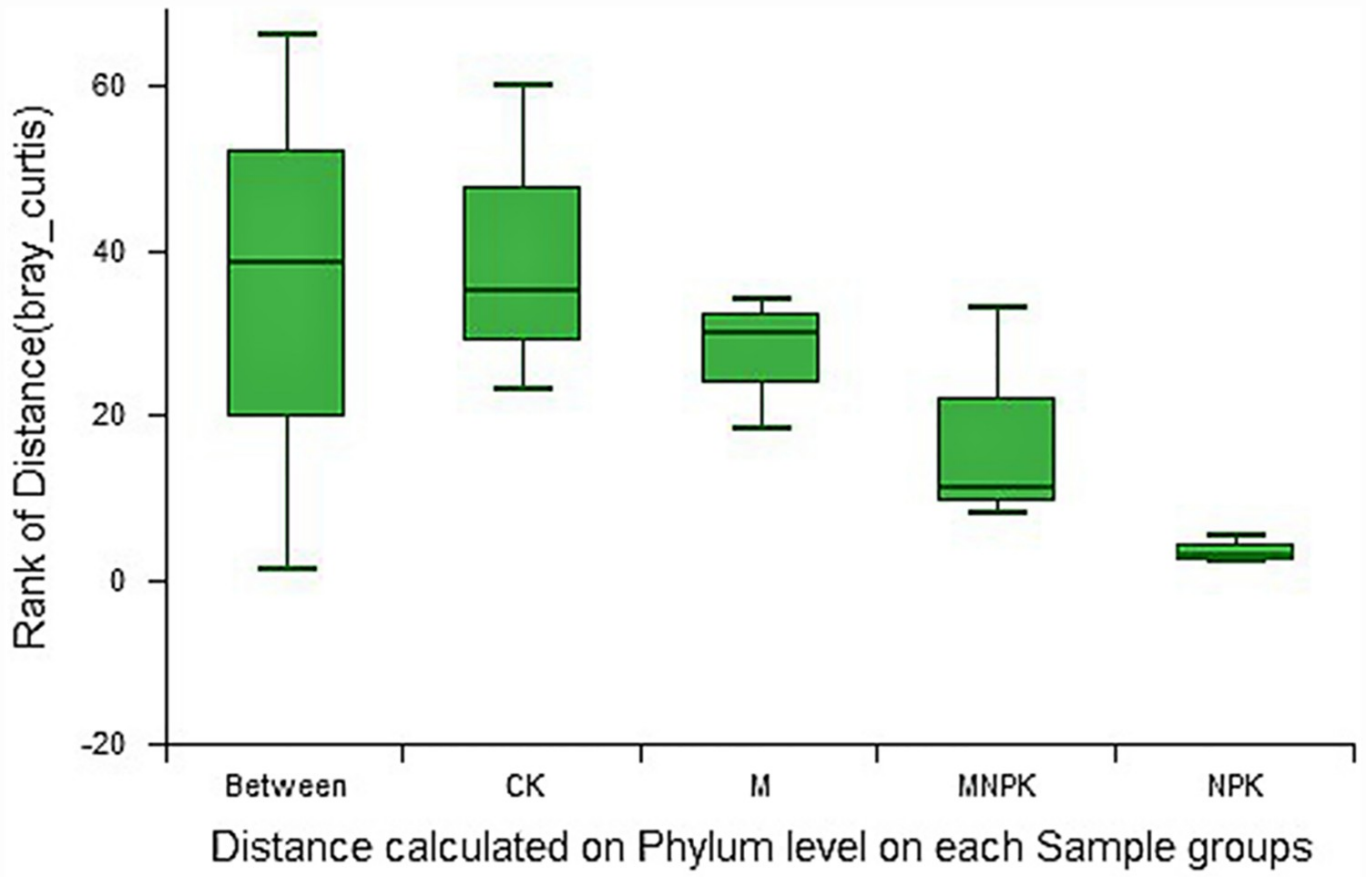
Analysis of similarities under different treatments at the Phylum level. The distance along the x-axis is the rank of distance value within or between groups. “Between” represents the distance value of the difference between the groups; group names represent the difference distance value within each group. The Y-axis represents the Bray-Curtis dissimilarity.

**Fig 3:**
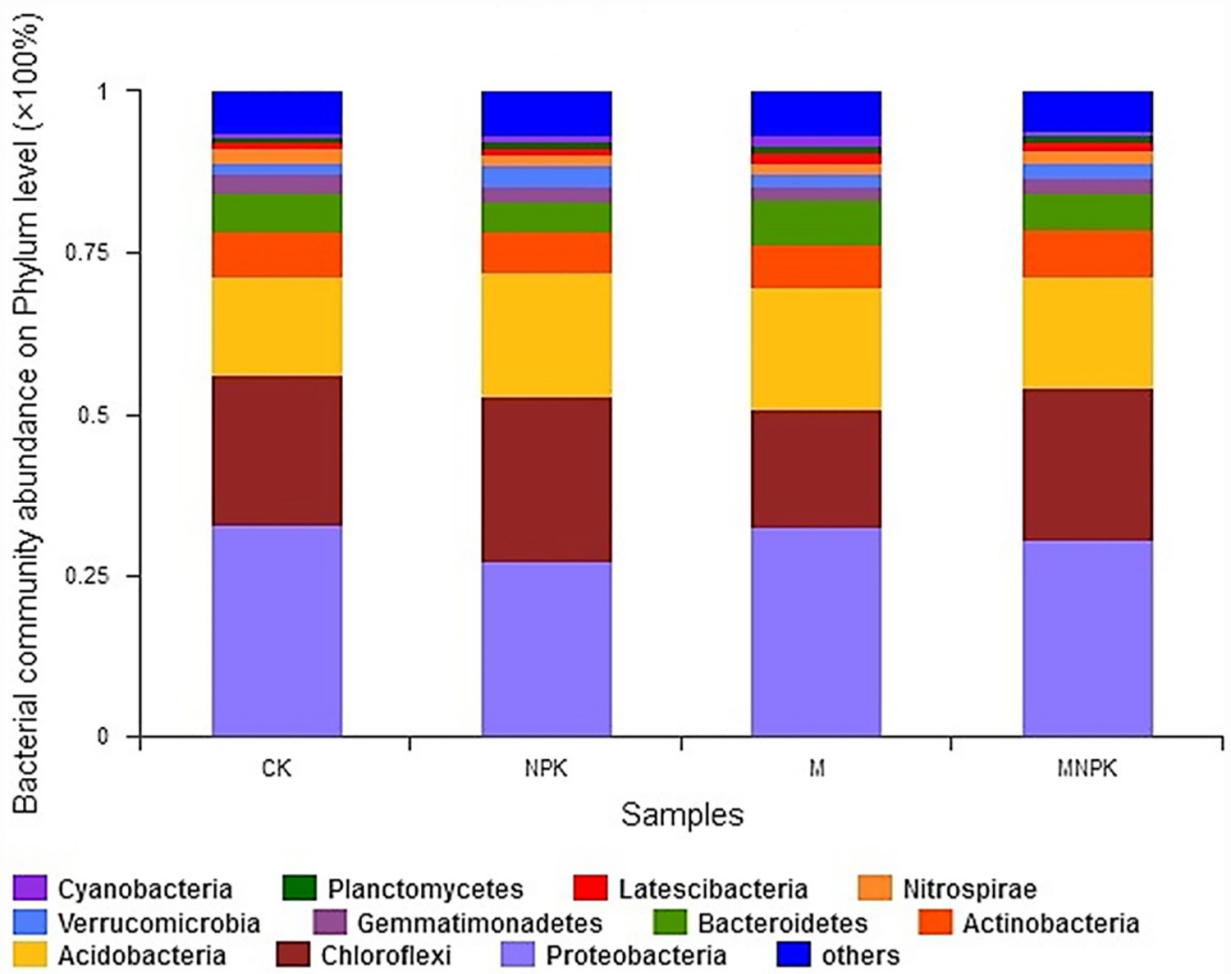
Bacterial community composition on the Phylum level. The % abundances of different bacterial phyla in each treatment group are shown in different colors.

The similarities analysis shows that differences between treatments were greater than differences within the groups, validating the results (F = 3.06, P < 0.05) (Fig 2 and S2 Table). Bacteria were mainly distributed in eleven phyla, including *Proteobacteria*, *Chloroflexi*, *Acidobacteria*, *Actinobacteria*, *Bacteroidetes*, and *Gemmatimonadetes*, which accounted for more than 85% of the bacterial community. The “Others” group, which made up 5% of the bacterial community, consisted of phyla with abundance of less than 1% of the bacterial population (Fig 3). Table 4 shows the differences of eleven phyla between the different fertilization treatments, among which the relative abundance of *Chloroflexius* (F = 5.38, P < 0.05), *Bacteroidetes* (F = 5.09, P < 0.05), and *Gemmatimonadetes* (F = 5.44, P < 0.05) were significantly different between groups.

**Table 4:** Bacterial relative abundance on the Phylum level under different fertilization treatment (%).

Phylum	Treatment			
	CK	NPK	M	MNPK
*Proteobacteria*	32.22 ± 5.72 a	27.24 ± 1.78 a	32.63 ± 3.81 a	30.74 ± 0.13 a
*Chloroflexi*	23.64 ± 3.76 a	25.41 ± 0.42 a	18.29 ± 1.93 b	23.29 ± 1.66 a
*Acidobacteria*	15.39 ± 2.56 b	19.51 ± 1.32 a	18.77 ± 1.97 ab	17.42 ± 0.89 ab
*Actinobacteria*	7.26 ± 0.67 a	6.29 ± 0.72 a	6.76 ± 0.71 a	7.26 ± 0.94 a
*Bacteroidetes*	5.70 ± 0.70 ab	4.44 ± 0.64 b	6.96 ± 0.56 a	5.68 ± 1.13 ab
*Gemmatimonadetes*	3.04 ± 0.73 a	2.54 ± 0.17 ab	1.79 ± 0.19 b	2.24 ± 0.08 b
*Verrucomicrobia*	1.67 ± 0.37 b	3.11 ± 0.44 a	2.06 ± 0.51 ab	2.28 ± 0.85 ab
*Nitrospirae*	2.34 ± 0.35 a	1.71 ± 0.09 b	1.83 ± 0.09 ab	1.96 ± 0.37 ab
*Latescibacteria*	1.09 ± 0.37 a	1.21 ± 0.17 a	1.53 ± 0.32 a	1.39 ± 0.34 a
*Planctomycetes*	0.76 ± 0.15 b	0.94 ± 0.02 ab	1.20 ± 0.39 a	1.02 ± 0.10 ab
*Cyanobacteria*	0.48 ± 0.19 b	0.94 ± 0.48 ab	1.52 ± 0.63 a	0.67 ± 0.26 b

Notes: Different small letters indicate significant differences (P < 0.05) within the same row. CK: no fertilization; NPK: chemical fertilizer; M: organic fertilizer; MNPK: constant organic-inorganic fertilizer. (n = 3, mean ± SE)

### Principal component analysis of bacterial community

**Fig 4:**
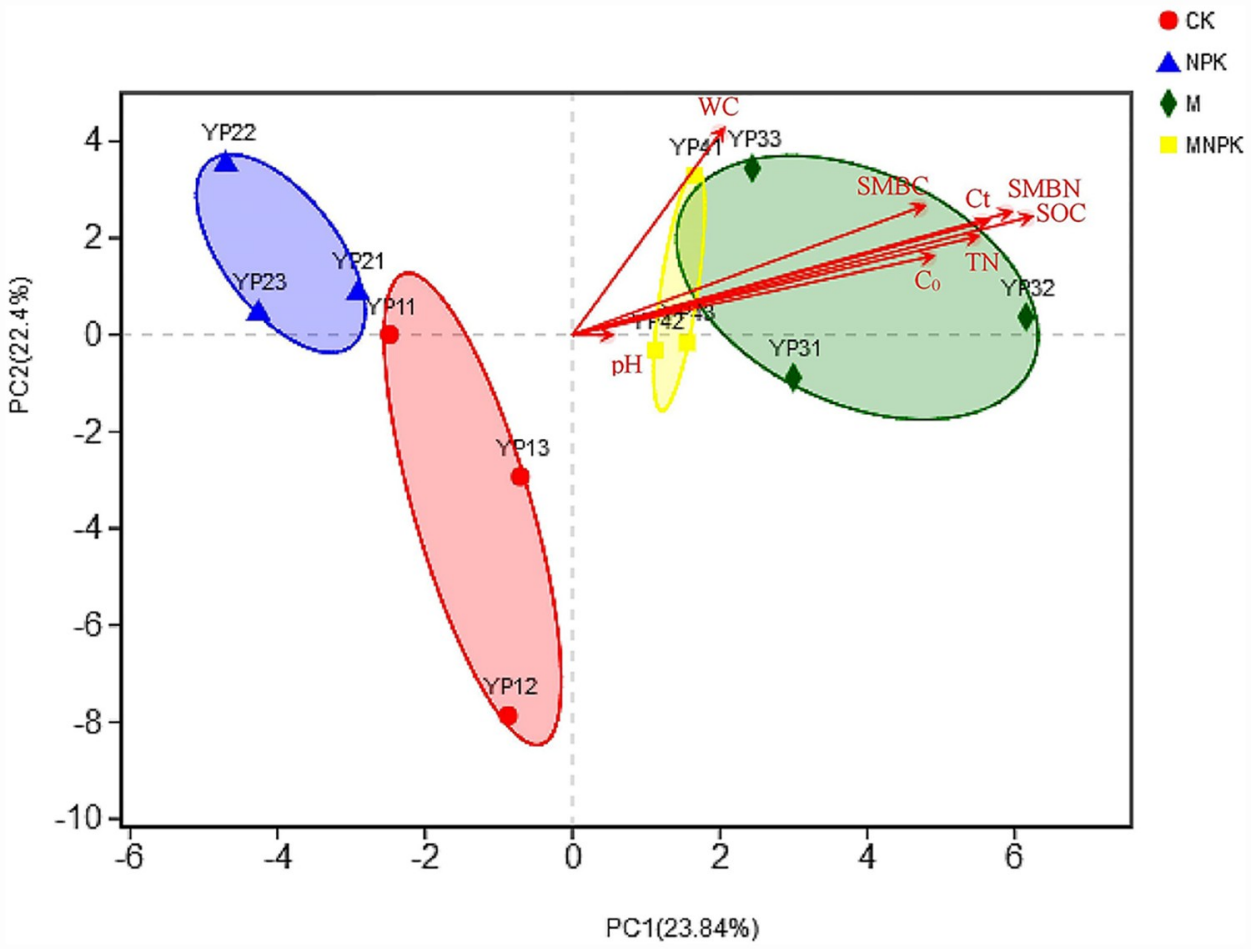
Principal component analysis of bacterial community composition and environmental factors on the Phylum level.

This study has done a principal component analysis between bacterial community composition and environmental factors based the Phylum level (Fig 4 and S2 Table). M and the MNPK treatments were close to each other, with some overlap. CK treatment and NPK treatment clustered separately. This indicates that there were significant differences in the composition of the microbial communities. The interpretation of the results for the PC1 axis and the PC2 axis was 23.84% and 22.4%, respectively. The length of the environmental factor vector in this study indicated the affect degree on species composition. The results showed that the contents of SOC (r = 0.8678, P < 0.01), SMBN (r = 0.8388, P < 0.01), C_t_ (r = 0.7911, P < 0.01), TN (r = 0.7594, P < 0.05), and SMBC (r = 0.7068, P < 0.05) were significantly correlated with the change in bacterial community in each sample.

### Correlation between microbes and environmental variables and mineralization parameters

**Fig 5:**
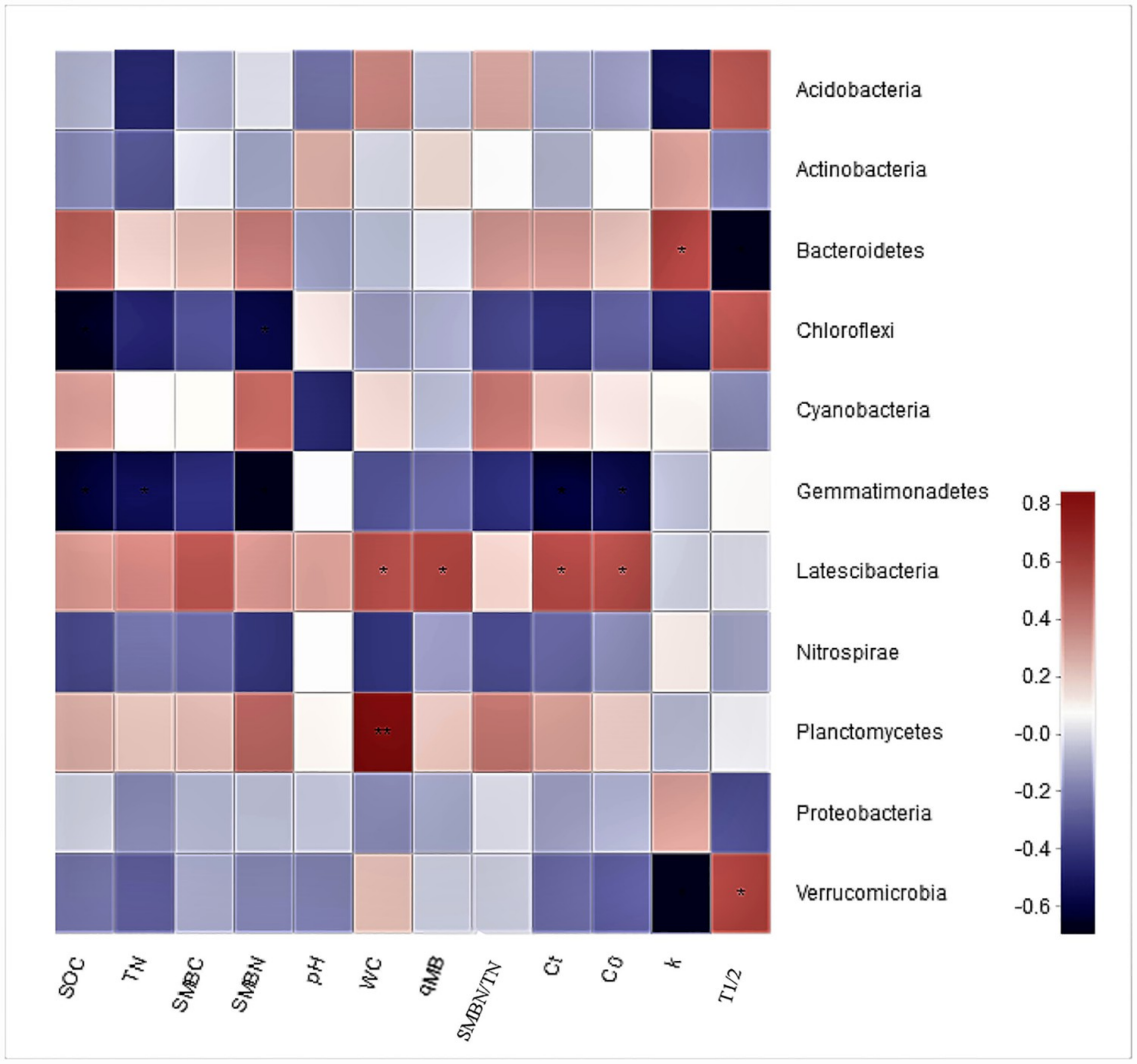
Pearson correlation heatmap of microbes and environmental variables and mineralization parameters. The X-axis and Y-axis represent the environmental factors and bacterial species, respectively. Color variations reflect positive (red) or negative (blue) correlation and the color depth indicates the level of positive or negative correlation, i.e. the darker the color, the better the correlation, as shown by the scale on the right. A P value less than 0.05 is denoted with *, a P value less than 0.01 is denoted with **.

*Chloroflexi* significantly negatively correlated with SOC and SMBN. *Bacteroidetes* showed a significant positive and negative correlation with k and T_1/2_, while the correlation between *Verrucomicrobia* and *Bacteroidetes* showed the opposite trend. *Gemmatimonadetes* showed significant negative correlation with SOC, TN, SMBN, C_t_, and C_0_. *Latescibacteria* significantly positively correlated with WC, qMB, C_t_, and C_0_. However, *Planctomycetes* only positively correlated with WC (Fig 5).

## Discussion

Fig 1 shows the cumulative mineralized carbon under different fertilization treatments during the culture period. In this study, chemical fertilizer did not significantly affect SOC content. This is consistent with the study by Li et al. in which the effects of chemical and organic fertilizer on soil properties after wheat crops were studied [29]. Li found no changes in carbon mineralization after chemical fertilization. In contrast, the application of organic fertilizer treatment in this study resulted in significantly increased carbon loss, as previously reported in other studies [30]. Fangueiro showed that addition of livestock slurry to soil resulted in large increases in CO_2_ emissions [30]. In our study, the total amounts of CO_2_ released after 30 d of incubation was significantly higher after organic fertilizer treatments (M and MNPK) compared to the chemical treatment. This data supports our hypothesis that organic fertilizer treatment can significantly increase organic carbon mineralization. The differences in organic versus chemical fertilizer effects may be due to inconsistent carbon inputs. Although the input of organic fertilizer directly increases the soil organic carbon content, organic fertilizers promote the growth of plants. The soil of chemical fertilizer application relies solely on the root residues and exudates of the crop to increase carbon input [31]. Therefore, the carbon source in the soil treated by organic fertilizer treatment was more abundant, which enhanced the microbial activity and accelerated the carbon pool mineralization process. It is worth noting that in the organic fertilizer treatment, carbon emission, and potential mineralized carbon content of MNPK treatment were higher than the M treatment, but the difference between treatments was not significant. This result highlights the promotion of carbon mineralization by high nitrogen fertilizer (the sum of N fertilizer that is applied in M treatment and NPK treatment) in the MNPK treatment compared to the M treatment, consistent with the study by Henrique et al. [31].

No significant difference was detected for the C_0_ value estimated by the first-order exponential model when soil was treated with chemical fertilizer (NPK). However, with organic fertilizer treatment (M and MNPK), the C_0_ increased significantly. Application of cow manure (M treatment) led to an increase in the mineralization rate constant (k) from 0.032 (CK) to 0.047 and consequently to a decrease in the half-life time (T_1/2_) from 21.7 to 14.8 d (Table 2). The trends were consistent with the investigation by Henrique et al. [31].

Our results show that long-term fertilization treatments had significant effects on microbial community composition (Fig 4). In particular, NPK treatment had significant effects on the relative abundance of a number of different bacteria phyla. Eo and Park [8] suggest that an imbalanced N-P-K ratio can drive changes in bacterial communities. Although soil was sampled only once in our study, the results support the conclusions. The long-term fertilization history and basic soil properties affected the microbial community composition. Jangid et al. [32] also showed that agricultural management practices, especially fertilizer treatments, over a long time, significantly alter the soil microbial community.

SOC and SMBN had the greatest impact on community composition, followed by C_t_, TN and SMBC (Fig 4). In our study, the 22 years of chemical fertilizer and organic fertilizer may have induced different soil carbon nitrogen conversion ratios, resulting in altered microbial community composition. The significant effects of nitrogen content on the microbial community is in agreement with other studies. For example, EO and Park [8] showed that N has significant effects on the bacterial community.

For soil organic carbon mineralization, the mineralization process is directly governed by the interactions between the amount of microbial biomass, microbial community structure, substrate quality and availability, soil properties, and microclimates [33]. In our study, the soil samples from all treatments were incubated at the same temperature and humidity, and thus, temperature and humidity had no influence on differences in C mineralization. However, the fertilizer treatments changed soil properties and, thus, may have indirectly changed C mineralization. The influence of microbial communities and soil properties on C mineralization was thus analyzed. The results of this study showed that more than 95% of the community composition in all treatments was similar between treatments (Fig 3 and Table 4). The three dominant bacteria in all treatments were *Proteobacteria*, *Chloroflexi*, and *Acidobacteria*, These 3 bacterial species were not associated with C mineralization changes (Fig 5). The non-dominant bacteria *Gemmatimonadetes* and *Latescibacteria* were significantly negatively correlated and positively correlated with C mineralization and soil properties, respectively, which was in accordance with our second hypothesis. Therefore, microbial abundance of several non-dominant bacterial species contributed more to the differences in C mineralization than the overall microbial community distribution. These results are in agreement with Kemmitt et al. [34], who also indicate that the composition of the soil microbial biomass does not regulate mineralization of native soil organic matter. Thus, soil microbial communities may be functionally redundant with respect to native organic C mineralization.

One drawback of this study was that we only sampled the soil during one period. Jangid et al. [32] demonstrated that seasons significantly affected bacterial composition, diversity, and abundance, and total microbial biomass was increased in winter; thus, sampling soil in different seasons may give different results. In this study, samples were collected in late October, after the rice harvest. Rice grows in April and is harvested in mid-to-late October in every year. During the rest of the year (approximately 6 months), the soil is fallow. At this stage, the surface soil changes from anaerobic to aerobic respiration, and soil moisture is not a dominant factor. We studied soil organic carbon mineralization and the microbial community in this context. Thus, our data elucidate changes in the soil only during this fallow period and can be the basis for soil management while the soil is resting. In addition, each of the 4 treatment areas were equally divided into 3 sections (67 m^2^). Samples were collected from 5 evenly spaced spots from each section, and then combined to get a representative sample. Three soil samples were collected from each treatment area. Consequently, there were a total of 60 points at which samples were collected. Thus, we feel that samples were representative of the soil during this fallow period. Despite the drawback of only one sampling period, the conclusions are still supported by the data presented in this study.

## Conclusion

Long-term fertilization influenced soil properties, microbial community, and C mineralization, and the differences were most obvious with constant organic-inorganic fertilizer treatment. Constant organic-inorganic fertilizer treatment significantly altered basic soil properties and increased organic C accumulation mineralization, potential mineralized C content, and turnover rate constant. We conclude that the difference in abundance of several bacteria influenced C mineralization, not the composition of soil microbial communities. In addition, SOC and SMBN were the most important contributors to the variance in microbial community composition. The non-dominant bacteria *Gemmatimonadetes* and *Latescibacteria* were significantly negatively correlated and positively correlated with C mineralization, which determined the difference in C mineralization.

Overall, our results demonstrate that long term fertilization influences the microbial community. Microbes are the main players in soil biochemical processes, and their abundance, activity, and composition are often closely related to soil organic carbon mineralization processes. Thus, understanding the response of the microbial community in different soil types is crucial in understanding the impact of long-term fertilization on the ecosystem.

## Supporting information

S1 Table Cumulative mineralization of soil organic carbon for 30 days and variance analysis. (XLS)

S2 Table Environmental factors data and operational taxonomic unit (OTU) based Phylum level. (XLS)
