# Correction: Effects of long-term fertilization on soil organic carbon mineralization and microbial community structure

**DOI:** 10.1371/journal.pone.0216006

**Published:** 2019-04-19

**Authors:** Zhen Guo, Jichang Han, Juan Li, Yan Xu, Xiaoli Wang, Yu Li

Dr. Yu Li should be included in the author byline. They should be listed as the sixth author, and their affiliation is **6**: Field Monitoring Experimental Station for Cultivated Land Preservation and Agro-environment in Guizhou, Ministry of Agriculture, Guiyang 550006, China. The contributions of this author are as follows: Provision of resources.

The correct citation is: Guo Z, Han J, Li J, Xu Y, Wang X, Li Y (2019) Effects of long-term fertilization on soil organic carbon mineralization and microbial community structure. PLoS ONE 14(1): e0211163. https://doi.org/10.1371/journal.pone.0211163
